# Pharmacovigilance in juvenile idiopathic arthritis patients treated with biologic or synthetic drugs: combined data of more than 15,000 patients from Pharmachild and national registries

**DOI:** 10.1186/s13075-018-1780-z

**Published:** 2018-12-27

**Authors:** Joost Swart, Gabriella Giancane, Gerd Horneff, Bo Magnusson, Michael Hofer, Еkaterina Alexeeva, Violeta Panaviene, Brigitte Bader-Meunier, Jordi Anton, Susan Nielsen, Fabrizio De Benedetti, Sylvia Kamphuis, Valda Staņēviča, Maria Tracahana, Laura Marinela Ailioaie, Elena Tsitsami, Ariane Klein, Kirsten Minden, Ivan Foeldvari, Johannes Peter Haas, Jens Klotsche, Anna Carin Horne, Alessandro Consolaro, Francesca Bovis, Francesca Bagnasco, Angela Pistorio, Alberto Martini, Nico Wulffraat, Nicolino Ruperto, for the Paediatric Rheumatology International Trials Organisation (PRINTO), BiKeR and the board of the Swedish Registry

**Affiliations:** 1 Department of Pediatric Immunology and Rheumatology, Wilhelmina Children’s Hospital, Lundlaan, 6 PO box 85090, Utrecht, The Netherlands; 2 IRCCS Istituto Giannina Gaslini, Clinica Pediatrica e Reumatologia, PRINTO, Via Gaslini, 5, 16147 Genoa, Italy; 3 Asklepios Clinic Sankt Augustin, Arnold-Janssen strasse 29, Sankt Augustin, Germany; 4 Department of Pediatric and Adolescents medicine, Medical faculty, University Hospital of Cologne, Kerpener Str. 62, Cologne, Germany; 5 Karolinska University Hospital, Pediatric Rheumatology Unit, Stockholm, Sweden; 6 Unité Romande d’Immuno-Rhumatologie Pediatrique/Centre Hospitalier Universitaire Vaudois (CHUV), Pediatrie, University of Lausanne, Av Bugnon 46, Lausanne, Switzerland; 7 University Hospital of Geneva, Geneva, Switzerland; 8 Federal State Autonomous Institution “National Medical Research Center of Children’s Health” of the Ministry of Health of the Russian Federation, LOMONOSOVSKIJ PR-T,2/62, Moscow, Russia; 9 Federal State Autonomous Educational Institution of Higher Education I.M. Sechenov First Moscow State Medical University of the Ministry of Health of the Russian Federation, Moscow, Russia; 10 Vilnius University, Clinic of Children’s Diseases, Vilnius, Lithuania and Children’s Hospital, Affiliate of Vilnius University Hospital Santariskiu Klinikos, Santariskiu, 4, Vilnius, Lithuania; 11 Université Paris-Descartes, Institut IMAGINE, Centre de référence national pour les Rhumatismes inflammatoires et les maladies Auto-Immunes Systémiques rares de l’Enfant (RAISE), Unité d’Immunologie, Hématologie et Rhumatologie Pediatrique, Hôpital Necker-Enfants Malades, Assistance Publique Hôpitaux de Paris, 149 Rue De Sevres, Paris, France; 12 Hospital Sant Joan de Déu, Universitat de Barcelona, Unidad de Reumatología Pediátrica, Esplugues de Llobregat, Passeig Sant Joan de Deu 2, Barcelona, Spain; 13 Juliane Marie Centret, Rigshospitalet, Paediatric Rheumatology Unit, Blegdamsvej 9, Copenhagen, Denmark; 14 Division of Rheumatology, IRCCS Ospedale Pediatrico Bambino Gesù, Piazza S. Onofrio, 4, Rome, Italy; 15 Sophia Children’s Hospital, Department of Paediatric Rheumatology, Erasmus University Medical Centre, Dr Molewaterplein 60, Rotterdam, The Netherlands; 16 Department of Rheumatology, Erasmus University Medical Centre, Rotterdam, The Netherlands; 17 Riga Stradins University, Department of Pediatrics, Children University Hospital, Vienibas gatve 45, Riga, LV Latvia; 18 Hippokration General Hospital, First Department of pediatrics, Thessaloniki University School of Medicine, Konstantinoupoleos 49, Thessaloniki, Greece; 19 Alexandru Ioan Cuza University of Iasi, V. Lupu str.nr. 62, Iasi, Romania; 20 Aghia Sophia Childrens Hospital, First Department of Pediatrics, University of Athens Medical School, Thivon 1, Athens, Greece; 21 German Rheumatism Research Centre, Berlin, Germany; 22 Charité University Medicine, Charitéplatz 1, Berlin, Germany; 23 Hamburg Centre for Pediatric and Adolescent Rheumatology, Dehnhaide 120, Hamburg, Germany; 24 German Center for Pediatric and Adolescent Rheumatology, Deutsches Zentrum für Kinder- und Jugendrheumatologie, Zentrum für Schmerztherapie junger Menschen, Gehfeldstrasse 24, Garmisch-Partenkirchen, Germany; 25 IRCCS Istituto Giannina Gaslini, Clinica Pediatrica e Reumatologia, via Gaslini 5, Genoa, Italy; 26 Dipartimento di Neuroscienze, Riabilitazione, Oftalmologia, Genetica e Scienze Materno-Infantili (DiNOGMI), Università degli Studi di Genova, Genoa, Italy; 27 IRCCS Istituto Giannina Gaslini, Servizio di Epidemiologia e Biostatistica, via Gaslini 5, Genoa, Italy; 28 IRCCS Istituto Giannina Gaslini, Direzione Scientifica, via Gaslini 5, Genoa, Italy

**Keywords:** Juvenile idiopathic arthritis, Registry, Safety, Adverse events, Methotrexate, Biologics

## Abstract

**Background:**

The availability of methotrexate and the introduction of multiple biological agents have revolutionized the treatment of juvenile idiopathic arthritis (JIA). Several international and national drug registries have been implemented to accurately monitor the long-term safety/efficacy of these agents. This report aims to present the combined data coming from Pharmachild/PRINTO registry and the national registries from Germany (BiKeR) and Sweden.

**Methods:**

Descriptive statistics was used for demographic, clinical data, drug exposure, adverse events (AEs) and events of special interest (ESIs). For the Swedish register, AE data were not available.

**Results:**

Data from a total of 15,284 patients were reported: 8274 (54%) from the Pharmachild registry and 3990 (26%) and 3020 (20%) from the German and the Swedish registries, respectively. Pharmachild children showed a younger age (median of 5.4 versus 7.6 years) at JIA onset and shorter disease duration at last available visit (5.3 versus 6.1–6.8) when compared with the other registries. The most frequent JIA category was the rheumatoid factor–negative polyarthritis (range of 24.6–29.9%). Methotrexate (61–84%) and etanercept (24%–61.8%) were the most frequently used synthetic and biologic disease-modifying anti-rheumatic drugs (DMARDs), respectively. There was a wide variability in glucocorticoid use (16.7–42.1%). Serious AEs were present in 572 (6.9%) patients in Pharmachild versus 297 (7.4%) in BiKeR. Infection and infestations were the most frequent AEs (29.4–30.1%) followed by gastrointestinal disorders (11.5–19.6%). The most frequent ESIs were infections (75.3–89%).

**Conclusions:**

This article is the first attempt to present a very large sample of data on JIA patients from different national and international registries and represents the first proposal for data merging as the most powerful tool for future analysis of safety and effectiveness of immunosuppressive therapies in JIA.

**Registry registration:**

The Pharmachild registry is registered at ClinicalTrials.gov (NCT01399281) and at the European Network of Centres for Pharmacoepidemiology and Pharmacovigilance (ENCePP) (http://www.encepp.eu/encepp/viewResource.htm?id=19362). The BiKeR registry is registered at ENCePP (http://www.encepp.eu/encepp/viewResource.htm?id=20591).

**Electronic supplementary material:**

The online version of this article (10.1186/s13075-018-1780-z) contains supplementary material, which is available to authorized users.

## Background

Juvenile idiopathic arthritis (JIA) [1] is the most common chronic pediatric rheumatic disease and an important cause of short- and long-term disability and quality-of-life impairment [2–8]. Although none of the available drugs for JIA has curative potential, prognosis has greatly improved as the result of substantial progress in disease management with the introduction of biologics. Despite the good efficacy results of all phase III trials on biologic agents, the long-term safety profile needs to be further characterized. For example, spontaneous reporting from countries with a low incidence of tuberculosis suggested that tuberculosis might be problematic in patients treated with biologics [9]. In August 2009, the US Food and Drug Administration (FDA) announced through a boxed warning that an increased risk of certain cancers in children might occur, and labeling for the tumor necrosis factor (TNF) blocker products was updated [10–14]. A Cochrane review from February 2011 compared the adverse events (AEs) of biologics and concluded that there is an urgent need for more research regarding their long-term safety of different biologics [15]. The availability of a large observational international and national registry could enable clinicians and regulatory agencies to properly monitor the long-term or rare safety events and effectiveness of these agents in the relatively low prevalent JIA.

The aim of this project is to present the combined data of the “Pharmacovigilance in JIA patients treated with biologic agents and/or MTX” (Pharmachild) international registry and two consenting JIA national registries: the “Biologics in Pediatric Rheumatology Registry” (BiKeR) from Germany and the JIA registry from Sweden. The secondary goal was to test a sharing system for future merging of data to address specific JIA scientific and clinical questions.

## Methods

### Description of registries

#### The Pharmachild registry

Pharmachild is an observational international registry that started in 2011 with European Union initial funding support and that enrolled children from member centers of the Paediatric Rheumatology International Trials Organisation (PRINTO) [16].

Inclusion criteria were children with JIA as per International League of Associations for Rheumatology (ILAR) criteria [17] receiving biologics or other synthetic disease-modifying anti-rheumatic drugs (DMARDs) as per physician decision. The registry contains two specific populations. The first is a retrospective cohort of all patients under treatment or previously treated with DMARDs by one-time clinical chart revision of safety events and complete drug exposure since disease onset to last available follow-up. The second is a prospective cohort including all cases newly treated with DMARDs since enrollment in the registry and cases still under treatment with any drug. To avoid selection bias, each center performed a census for all patients previously treated with DMARDs at that specific center, used as the reference to evaluate the enrollment capability. In a second step, the center entered retrospective data, considered successful if they retrieved at least 70% of the patients listed in the census. Finally, in a third step, the prospective data collection started.

Data collection included full and complete details for ILAR classification criteria; demographic, clinical, and laboratory information; and efficacy (only for the prospective cohort) and safety data on a long-term basis. Centers reported the whole drug exposure of the patient along with dates of start and discontinuation of the drug, dosages, route of administration, reasons for discontinuation, and possible correlation with the AEs. All the AEs of at least moderate/severe/very severe intensity and serious AEs, using the latest release of the Medical Dictionary for Regulatory Activities (MedDRA) dictionary, were reported; mild intensity was reported only for those AEs which did not resolve and require a follow-up report. Some AEs were classified as by consensus of PRINTO members as events of special interest (ESIs).

Efficacy data were collected in the prospective cohort through the JIA core set measures with whole joint count [18], the disease activity status measured through the Juvenile Arthritis Disease Activity Score (JADAS), and the annual evaluation of damage through the Juvenile Arthritis Damage Index (JADI) [19] and of growth and pubertal development and key information on imaging and bio-specimen local collection. As patient-reported outcome (PRO), families completed online the Juvenile Arthritis Multidimensional Assessment Report (JAMAR) [20] before the scheduled clinic visit or in the hospital (on tablets or paper) in order to provide key notes to the treating physician before the clinical examination.

**Fig. 1:**
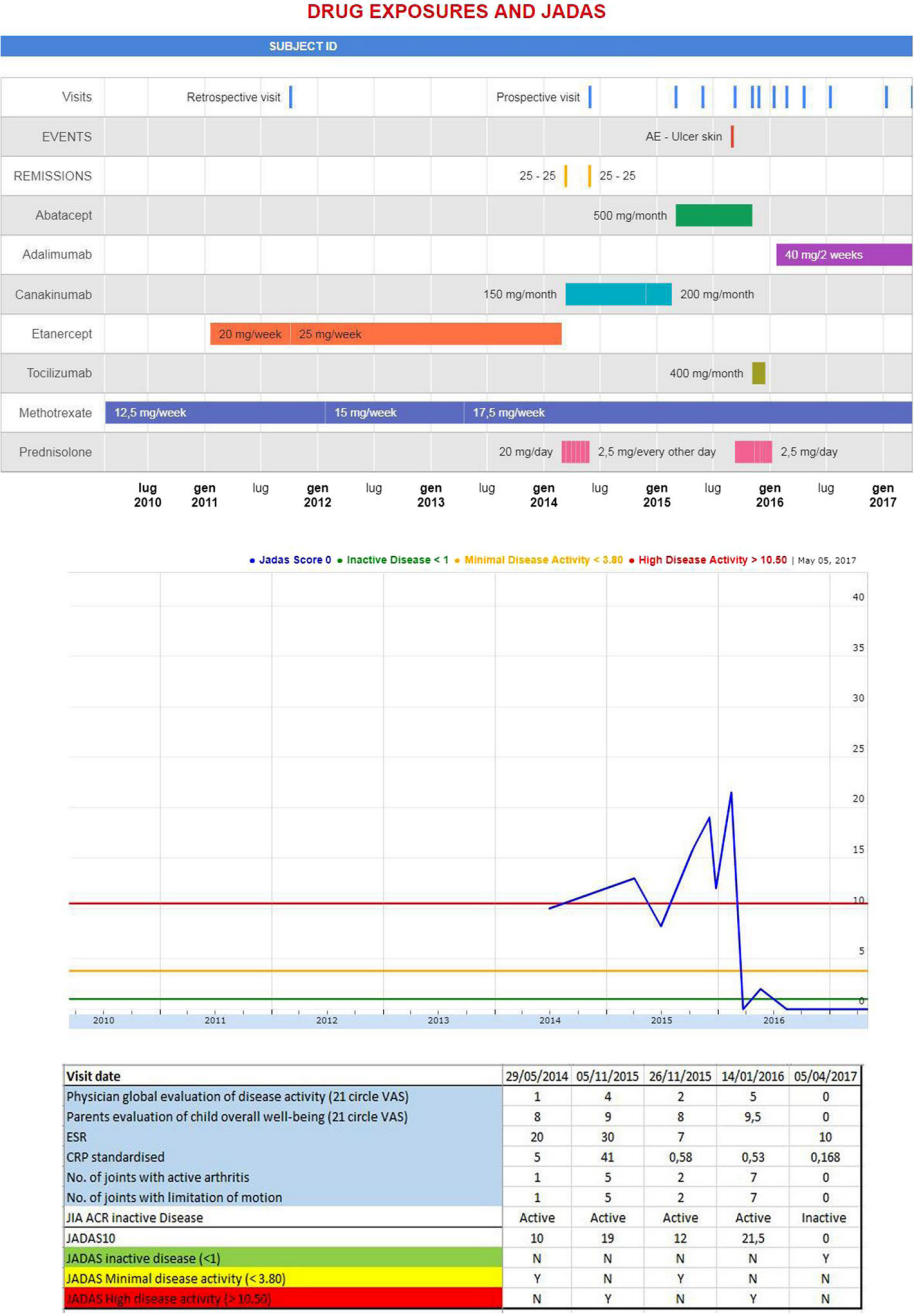
Pharmachild graphical depiction over time of the key efficacy and safety data. Drug exposure and adverse events are represented in parallel to Juvenile Arthritis Disease Activity Score (JADAS) pattern. The Excel spreadsheet with all the data could be downloaded automatically by all participating centers. In the figure, an example of a patient from an Italian center is presented

The system also provided data on drug exposure and occurrence of AEs (Fig. 1) as a tool to discuss the health status of a patient with the family.

Data collection was performed online via the secured PRINTO website on a dedicated server with a username and timely password on an https-encrypted platform. English was the official language used for all forms completed by the physicians, and the PROs were available in the appropriate language spoken by parents/patients. The web system was designed to be user-friendly, modular, and upgradable. During data entry, several hundred automatic checks were in place to ensure data quality and consistency. In particular, safety events were checked for accuracy by PRINTO-certified MedDRA coders, which could go back to the center with electronic query tickets in case of missing or unclear information (Additional file 1). A designated pediatric rheumatologist acted as medical monitor (JS) by performing an electronic check and revision of the AEs and ESIs; in addition, for some ESIs (for example, infection), adjudicating committees were in place.

#### The BiKeR registry

The BiKeR registry in Germany focused, from 2001, on AEs and efficacy data in patients treated with etanercept (ETN), the first biologic licensed in Germany [21]. From that time, surveillance was extended to all biologics approved for JIA [22–24]. Information on biologics not approved for JIA was also collected for such patients who have been admitted for an approved biologic if patients were switched. The BiKeR registry was founded by pharmaceutical companies with independent bilateral contracts. BiKeR was approved by the ethics committee of the physician board of Aerztekammer Nordrhein, Düsseldorf. The BiKeR registry includes about 80 study sites and since its inception has followed more than 4000 patients in Germany and Austria who were 2 to 18 years old and who meet the ILAR criteria for JIA. Written consent was obtained from patients and parents and repeated if the patient became an adult. Only pseudonymized data were collected.

Patient demographic characteristics, disease history, and previous treatments are documented at the time of patient enrollment. Details about relevant treatment and reasons for discontinuation, concomitant therapy, disease activity, and AEs are prospectively collected by using standard case report forms (CRFs) at the start of treatment, at months 3 and 6, and every 6 months thereafter. Safety was analyzed on the basis of AE reporting. All reported AEs defined as any untoward medical occurrence in a subject administered a pharmaceutical product, even without a causal relationship with the treatment, were analyzed. Serious AEs and ESIs were defined as in Pharmachild. Onsite monitoring is performed in selected larger centers covering about 80% of admitted patients. In 2005, the registry was extended to include a control group of 1500 biologic-naïve JIA patients who started with the synthetic DMARD such as MTX to enable comparison of patients exposed to biologics with unexposed JIA cohorts [25, 26]. The “Juvenile arthritis MTX/Biologics long-term Observation” (JUMBO) was launched in 2007 to include data on long-term safety after transition to adulthood [27]. At present, 3990 patients are included in the JUMBO registry.

#### The Swedish registry

In 2009, the Swedish JIA registry began to follow all children on biologics and later expanded to all patients treated with or without DMARDs. Reports from care givers, patients, and medical records using JADAS, quality-of-life questionnaires, and arthritis-specific questions were included in the registry, which after 5 years includes 1700 children (60% of the total JIA population and above 90% of patients on cytokine modulators). Data on treatment as well as disease course and efficacy were included, while data on safety were not available [28].

#### Statistics

All registries and participating centers obtained approval from their respective ethics committee and obtained consent/assent based on existing national regulations.

Pharmachild, BiKeR, and the Swedish registries reported cumulative summary data into predefined spreadsheets in order to provide baseline descriptive statistics of demographic and clinical data. Safety data were available only for Pharmachild and BiKeR. ESIs common to the two registries are reported.

For qualitative data, frequencies (percentage) were reported, while quantitative data were expressed in terms of medians with first and third quartiles. No formal statistical comparisons were performed.

## Results

### Demographic characteristics and drug exposure

In Pharmachild, 11,796 patients in total were registered in the census registry as of January 2017 from 98 PRINTO centers in 32 countries. Clinical and safety data were provided for 8274 (70.1%) out of 11,796 patients belonging to 86 participating centers. Sixty out (61.2%) of eighty-six centers provided at least 70% safety data of their local JIA patients, and the median was 55 patients per center. Prospective data were collected for a total of 3070 patients.

Table 1 reports the demographic and clinical data for a total of 15,284 patients: 8274 (54.1%) from the Pharmachild registry and 3990 (26.1%) from the German BiKeR and 3020 (19.8%) from the Swedish registry. The patients included in the German and Swedish registries were not overlapping with those in Pharmachild since the registries were created in different periods and included data from different patients with the same disease.

**Table 1:** Demographic and clinical characteristics of the juvenile idiopathic arthritis patients from different registries

	Pharmachild *N* = 8274	BiKeR *N* = 3990	Sweden *N* = 3020
Number of countries	32	2#	1
Number of centers	86	72	33
Number of patients per center	55.5 (17–124)	10.5 (3–39.8)	52 (31–78)
Age at onset	5.4 (2.4–10.0)	7.6 (3.2–11.7)	7.6 (2.9–11.7)^1^
Age at JIA diagnosis	6.2 (2.8–10.9)	–	8.3 (3.5–12.8)^2^
Disease duration at last visit	5.3 (2.7–8.8)	6.1 (3.5–9.5)	6.8 (4.3–10.1)^3^
Female	5584 (67.5%)	2670 (66.9%)	1989 (65.9%)
Antinuclear antibodies (ANA)*	1767 (21.4%)	1900 (47.6%)	–
ILAR JIA category		4	5
Systemic	911 (11.0%)	267 (6.7%)	109 (4.7%)
Oligo	3071 (37.1%)	1215 (30.5%)	1148 (49.6%)
Oligo persistent	2011 (24.3%)	494 (12.4%)	–
Oligo extended	1060 (12.8%)	721 (18.1%)	–
Polyarticular RF^−^	2183 (26.4%)	1192 (29.9%)	568 (24.6%)
Polyarticular RF^+^	322 (3.9%)	243 (6.1%)	85 (3.7%)
Psoriatic arthritis	285 (3.4%)	296 (7.4%)	160 (6.9%)
Enthesitis-related arthritis	924 (11.2%)	649 (16.3%)	185 (8.0%)
Undifferentiated arthritis	578 (7.0%)	127 (3.2%)	58 (2.5%)

Data are medians (1st–3rd quartiles) or frequencies (percentages)

^*^ANA at least two consecutively positive determinations according to local standards

# Germany and Austria

^1^data available for 2477 subjects

^2^data available for 2197 subjects

^3^data available for 2479 subjects

^4^data available for 3989 subjects

^5^data available for 2313 subjects

Abbreviations: *BiKeR* Biologics in Pediatric Rheumatology Registry, *ILAR* International League of Associations for Rheumatology, *JIA* juvenile idiopathic arthritis, *RF* rheumatoid factor

Patients coming from the Pharmachild database showed a younger age (median of 5.4 years versus 7.6) at onset and shorter disease duration (5.3 versus 6.1–6.8) at the last available follow-up visit in comparison with the other registries. BiKeR reported a lower median number of children per center (10.5 versus 52–55.5). Antinuclear antibody (ANA) positivity was higher in BiKeR and missing in the Swedish registry.

The JIA category distribution differed among registries, but the most frequent JIA category was rheumatoid factor (RF)–negative polyarthritis (range of 24.6%–29.9%). The frequency of oligoarticular JIA was higher in the Swedish registry (49.6% versus about 30.5%–37.1% in the other two registries), while in BiKeR the frequencies of oligo- and poly-articular JIA RF-negative were similar (about 30%); Pharmachild depicted a higher frequency of systemic JIA (11% versus 4.7–6.7% in the German and Swedish registries, respectively).

Table 2 reports the number of patients who ever received a drug from onset to last available follow-up visit, with the corresponding days of drug exposure per medication from the first day of drug administration to the last available follow-up visit, excluding the days off therapy for any reason.

**Table 2:** Number of patients who ever received a drug from onset to last available follow-up visit, with the corresponding days of drug exposure per medication from the first day of drug administration to the last available follow-up visit

		Pharmachild N = 8274 Days of drug exposure	BiKeR N = 3990 Days of drug exposure	Sweden N = 3020 Days of drug exposure
DMARDs	Methotrexate	6963 (84.2%); 924 (449–1747)	3344 (83.8%); 494 (173–957)	1842 (61%); 1198 (555–2127)
	Sulfasalazine	861 (10.4%); 360 (143–730)	274 (6.9%); 174 (32–470)	95 (3%) 443 (132–1042)
	Cyclosporine	518 (6.3%); 616 (235–1358)	113 (2.8%); 186 (62–580)	16 (0.5%); 584 (250–1452)
	Leflunomide	372 (4.5%); 434 (182–888)	219 (5.5%); 267 (68–701)	2 (0.1%); 840 (511–1169)
	Hydroxychloroquine	279 (3.4%); 486 (202–1022)	106 (2.7%); 182 (1–535)	32 (1.1%); 957 (311–1612)
	Azathioprine	108 (1.3%); 439 (187–973)	155 (3.9%); 186 (26–494)	31 (1%); 1171 (340–2179)
	Thalidomide	35 (0.4%); 290 (85–665)	0	0
Systemic glucocorticoids		3299 (39.9%) 206 (67–648)	1680 (42.1%) 196 (81–449)	503 (16.7%) 91 (35–437)
Biologics	Etanercept	3600 (43.5%); 719 (300–1338)	2467 (61.8%); 489 (184–934)	726 (24%); 827 (341–1666)
	Adalimumab	1778 (21.5%); 442 (174–927)	810 (20.3%); 350 (117–755)	657 (21.8%); 701 (292–1604)
	Infliximab	705 (8.5%); 425 (160–951)	68 (1.7%); 213 (129–717)	189 (6.3%); 825 (328–1738)
	Tocilizumab	633 (7.7%); 351 (126–742)	281 (7%); 377 (127–730)	122 (4%); 660 (193–1353)
	Abatacept	420 (5.1%); 342 (156–715)	101 (2.5%); 190 (83–582)	80 (2.6%); 378 (164–1125
	Anakinra	339 (4.1%); 299 (94–837)	50 (1.3%); 304 (9–806)	48 (1.6%); 422 (144–836)
	Golimumab	161 (1.9%); 270 (106–623)	63 (1.6%); 344 (88–783)	93 (3.1%); 796 (370–1743)
	Canakinumab	145 (1.8%); 351 (133–1032)	39 (1%); 364 (214–733)	7 (0.2%); 654 (604–1654)
	Rituximab	103 (1.2%); 42 (24–87)	4 (0.1%); 15 (0–108)	20 (0.7%); 129 (15–1550)
	Certolizumab	33 (0.4%); 166 (106–309)	4 (0.1%); 49 (0–110)	8 (0.3%); 984 (714–1538)
	Other biologic agents	14 (0.2%); 217 (54–432)	4 (0.1%); 77 (25–149)	2 (0.1%); 325 (223–426)

Data are numbers of patients with frequencies (percentage), and medians and 1st–3rd quartiles of days of drug exposure

Abbreviations: *BiKeR* Biologics in Pediatric Rheumatology Registry, *DMARD* disease-modifying anti-rheumatic drug

There was a global trend to use MTX as a first-choice synthetic DMARD and ETN as a first-line biologic, but the Swedish registry used these drugs in a lower percentage of patients (MTX 61% versus about 84% in Pharmachild and BiKeR; ETN 24% versus 43.5% in Pharmachild and 61.8% in BiKeR). Despite the similar percentage of patients using these medications, children from BiKeR were exposed for a shorter period to the drugs compared with Pharmachild children, whereas the Swedish registry demonstrated a much longer drug exposure, with a wide range of variability among patients. Adalimumab, among the most frequently used biologics, was administered in similar percentages of patients among all three databases (about 21% of patients). Systemic steroids were used in similar percentages of patients and with the same drug exposure in BiKeR and Pharmachild, whereas the Swedish registry administered shorter cycles of steroids in a smaller number of patients (about 40% of patients in Pharmachild and BiKeR versus 16.7% in the Swedish registry).

### Safety data

Overall, the German registry showed a higher incidence of AEs but with lower intensity. In Pharmachild, 1599 (19.3%) of 8274 patients reported at least one moderate AE compared with 1747 (43.8%) of 3999 AEs of any intensity patients in BiKeR. Indeed, when the AEs of at least moderate intensity were compared between the two registries, the differences were less pronounced (18.5% for Pharmachild versus 10.2% in BiKeR). Serious AEs were present in 572 patients (6.9%) in Pharmachild versus 297 (7.4%) in BiKeR. Among them, 13 deaths were reported in Pharmachild and 3 in BiKeR mainly due to severe infections or malignancies or both.

Table 3 reports a total of 5173 AEs in Pharmachild and 5013 in BiKeR, according to the MedDRA dictionary divided by system organ class (SOC). Infection and infestations resulted as the most frequent SOC in Pharmachild and BiKeR (29.4% versus 30.1%, respectively) followed by gastrointestinal disorders (11.5% versus 19.6%) whereas all remaining SOCs occurred in less than 10% of the AEs. In Pharmachild, more injuries, poisoning, and complications and hematological and hepatobiliary disorders were reported compared with BiKeR, which showed more investigations, general disorders and administration site conditions, and neurological and immune system disorders. The numbers of uveitis, included in “Eye disorders” category, were comparable in the two registries (5.2% versus 6.2% in Pharmachild and BiKeR, respectively).

**Table 3:** Total number of adverse events by MedDRA system organ class ordered by decreasing frequencies

	Pharmachild *N* = 5173	BiKeR *N* = 5013
Infections and infestations	1523 (29.4%)	1509 (30.1%)
Gastrointestinal disorders	595 (11.5%)	984 (19.6%)
Injury, poisoning and procedural complications	325 (6.3%)	152 (3.1%)
Blood and lymphatic system disorders	291 (5.6%)	99 (2%)
Investigations	285 (5.5%)	377 (7.5%)
Eye disorders	270 (5.2%)	309 (6.2%)
Skin and subcutaneous tissue disorders	256 (4.9%)	217 (4.3%)
General disorders and administration site conditions	245 (4.7%)	410 (8.2%)
Hepatobiliary disorders	233 (4.5%)	24 (0.5%)
Surgical and medical procedures	209 (4.1%)	98 (2%)
Nervous system disorders	151 (2.9%)	227 (4.5%)
Musculoskeletal and connective tissue disorders	147 (2.8%)	138 (2.7%)
Respiratory, thoracic, and mediastinal disorders	112 (2.2%)	50 (1%)
Psychiatric disorders	105 (2.1%)	157 (3.1%)
Endocrine disorders	104 (2.0%)	6 (0.1%)
Metabolism and nutrition disorders	77 (1.5%)	34 (0.7%)
Renal and urinary disorders	66 (1.3%)	21 (0.4%)
Immune system disorders	33 (0.6%)	77 (1.5%)
Vascular disorders	30 (0.6%)	46 (0.9%)
Reproductive system and breast disorders	26 (0.5%)	13 (0.3%)
Congenital, familial, and genetic disorders	22 (0.4%)	9 (0.2%)
Cardiac disorders	19 (0.4%)	13 (0.3%)
Neoplasms benign, malignant, and unspecified (including cysts and polyps)	16 (0.3%)	29 (0.6%)
Ear and labyrinth disorders	13 (0.3%)	7 (0.1%)
Social circumstances	11 (0.2%)	0
Pregnancy, puerperium and perinatal conditions	9 (0.2%)	7 (0.1%)

Data are absolute numbers and frequencies (percentage)

Abbreviation: *BiKeR* Biologics in Pediatric Rheumatology Registry, *MedDRA* Medical Dictionary for Regulatory Activities

These results were confirmed by analyzing the distribution of AEs separately for the retrospective and the prospective visits. We identified a total of 1050 AEs extracted from the prospective visits, and 4123 events by the retrospective data, divided by SOC. In general, the hierarchy and frequency of AEs were similar, and infections and infestations were the most frequent events (Additional file 2).

Table 4 reports details for the 2022 and 1697 common ESIs in Pharmachild and BiKeR, respectively. The most frequent ESIs were infections, which were the most prevalent in both registries (75.3% versus 89% in Pharmachild and BiKeR, respectively), followed by blood cell–related ESIs. In Pharmachild, infusion/injection-related reactions were more frequent than in BiKeR (10.8% versus 1.4%).

**Table 4:** Total number of events of special interest ordered by decreasing frequencies

	Pharmachild *N* = 2022	BiKeR *N* = 1697
Infections:	1523 (75.3%)	1509 (89%)
Serious/targeted infections (Epstein–Barr virus, cytomegalovirus, papilloma virus, herpes zoster primary and reactivation, and opportunistic infections)	674 (33.3%)	171 (10.1%)
Tuberculosis	27 (1,3%)	0
Other infections	822 (40.6%)	1338 (78.8%)
Infusion/injection related reactions:	218 (10.8%)	24 (1.4%)
Infusion related reaction	144 (7.1%)	11 (0.6%)
Injection related reaction	74 (3.7%)	13 (0.8%)
Blood cell–related events of special interest (ESI):	188 (9.3%)	90 (5.3%)
Pancytopenia	6 (0.3%)	65 (3.8%)
Neutropenia	107 (5.3%)	14 (0.8%)
Macrophage activation syndrome	75 (3.7%)	11 (0.6%)
Aplastic anemia	0	0
Autoimmune ESI:	50 (2.5%)	50 (2.9%)
Inflammatory bowel disease (IBD)	21 (1.1%)	23 (1.3)
Other autoimmune diseases excluding IBD, uveitis, and demyelinisation disorders	18 (0.9%)	24 (1.4%)
Lupus erythematosus systemic/lupus-like syndrome	4 (0.2%)	1 (0.1%)
Optic neuritis	4 (0.2%)	0
Multiple sclerosis	2 (0.1%)	0
Demyelination	1 (0.05%)	2 (0.2%)
Malignancies:	16 (0.8%)	13 (0.8%)
Leukemias	3 (0.1%)	2 (0.2%)
Lymphomas	2 (0.1%)	5 (0.3%)
Hematopoietic neoplasms (excluding leukemias and lymphomas)	1 (0.05%)	2 (0.2%)
Neoplasm (other)	10 (0.5%)	4 (0.2%)
Others ESI:	27 (1.3%)	11 (0.6%)
Gastrointestinal (GI) ulcer/GI bleed/GI perforation	17 (0.8%)	4 (0.2%)
Pregnancy	9 (0.4%)	7 (0.4%)
Congestive heart failure	1 (0.05%)	0

Data are absolute numbers and frequencies (percentage)

There were 27 cases of tuberculosis reported in Pharmachild (52% from Asia, 37% from Europe, and 11% from the US) and none in BiKeR, whereas all serious/targeted infections were 674 (33.3%) and 171 (10.1%), respectively; 17 cases of tuberculosis were during biologic therapy, namely TNF inhibitors in 14 patients.

There were few cases of malignancies reported in either registry. Besides the reported cases of hematological malignancies in Table 4, in Pharmachild we could observe 10 additional cases (neoplasm others), represented for one third by hemangioma, and with the remaining patients suffering from thyroid cancer, cervix neoplasm, skin tumors, breast fibroadenoma, colon adenoma, and osteochondroma. The German registry reported in the same group similar malignancies, in particular of the genital apparatus (thyroid carcinoma, germ cell tumor, anaplastic ependymoma, and cervix dysplasia).

## Discussion

Since the 1990s, when the first immunomodulatory products for rheumatic diseases were introduced, the benefits of synthetic and biologic DMARDs became clear in the management of JIA. Currently, however, safety information for JIA is derived mainly from phase III clinical trials and more recent registries and administrative claims. Therefore, little information exists on the long-term safety of these agents. In 2009, a great scientific debate regarding the safety of TNF blockers started, prompting the FDA to issue a warning regarding a possible association between the use of TNF blockers and the development of lymphoma and other cancers in children and young adults with JIA [29]. Until now, owing to confounding factors such as the use of concomitant immunosuppressants, the effect of biological therapies on the risk to develop cancer or other risks such as infections in JIA is still controversial [30]. Literature has provided evidence that an increased risk of malignancy exists among children with JIA when compared with the general population, irrespective of medication use. Conversely, other studies have not confirmed these findings, highlighting the need of further studies to estimate this risk more accurately [11, 13, 31, 32]. In order to address this and other safety concerns more reliably, several methods for pharmacovigilance could be implemented, spanning from the results of phase II and III clinical trials to post-marketing passive reporting or from registries (not-for-profit or sponsored by pharmaceutical companies) [10, 33]. For this purpose, several registries have been created in the last decade; in particular, the national pediatric rheumatology societies in European countries and in North America initiated independent registries or registries in collaboration with pharmaceutical companies for the long-term evaluation of the safety and effectiveness mainly of biologic DMARDs [26, 28, 33–37]. Other research groups have concentrated their efforts on the analysis of insurance claims [30, 38]. PRINTO implemented Pharmachild in order to guarantee a critical mass of patients’ data and to provide systematically obtained evidence for provision of reliable scientific data for health professionals and health authorities. Aiming to avoid overlapping of data collection and to find an agreement on the proper way to share common data, a considerable number of European pediatric rheumatology societies (for example, in France, the Netherlands, Spain, and the Czech Republic primarily) agreed to use Pharmachild as their primary resource for data collection.

This article is the first attempt to present a very large sample of data on JIA patients from different registries, providing an overview on the baseline characteristics from international and national registries. This analysis highlights some differences but also similarities. An important difference that was observed was the higher frequency of AEs in the German BiKeR registry, even though associated with a lower intensity, which may reflect the different inclusion criteria of the two registries. Indeed, in Pharmachild, events of mild intensity, defined as transient or mild discomfort (<48 h) and no medical intervention/therapy required, are excluded. This difference is the trade-off implemented in Pharmachild in order to concentrate on more important safety events and facilitate data collection in everyday busy clinical practice.

Similarities among registries regarding therapies and AEs could be identified. MTX was the most used synthetic DMARD. ETN was the most frequently used biologic agent in all registries considered, followed by adalimumab. Drug exposure differed the three databases; in BiKeR, it was lower for almost all the medications; in the Swedish registry, it was much longer and had a wider range of exposure variability despite the similar disease duration. The relatively high rate of ETN use in the BiKeR registry might be explained by the fact that this registry originally started as a registry for this specific drug, when ETN was the only approved biological drug in pediatric rheumatology and then extended to other medications after their approval. However, in more recent years in BiKeR, ETN is the first biologic in about two thirds of patients with non-systemic JIA. Systemic steroids were used much less in Sweden and for shorter periods and this was maybe due to the lower incidence of systemic JIA. In regard to ESIs, infections were the most common event in both Pharmachild and BiKeR registries whereas malignancies were reported in a limited number of patients. The overall frequencies of the different AEs and ESIs were similar between Pharmachild and BiKeR. The major differences when comparing Pharmachild with BiKeR were higher frequencies of tuberculosis infection and infusion/injection-related reactions in the first for a possible interviewer bias elicited by the Pharmachild CRFs, which explicitly focus the attention of the clinicians to these AEs. The difference in the rate of tuberculosis infections may also reflect a different risk among European countries and the need for higher awareness of this problem in some regions.

Next to reporting baseline data from a large sample of patients with JIA , this study could not merge individual patient data because of the lack of homogeneous information. Therefore, it can be seen as a practical proposal for future studies that involve data merging. We propose a three-step procedure for future studies. In step 1, the CRFs of the different registries should be compared highlighting the similarities and differences. Step 2 will verify the database technical characteristic (for example, Sql server version 2005 and Access 2010) and the field coding (for example, gender, int, 1 = male; 2 = female). The third step related to the individual patient’s data merging. An Excel spreadsheet with the data specifications related to a specific article will be shared with the participating registries. Each registry will have to add its own data related to the project. The coordinator of the project will merge the individual patients’ data after proper coding transformation. A census (for example, few demographic data of all patients in the registry) will be provided by each registry as a preliminary step to check for a potential selection bias. The coordinator will then prepare an additional spreadsheet to highlight the important missing information (query log) to be resolved in a timely manner in order to proceed with the final analysis and drafting of the article. The entire procedure may meet some obstacles due to the lack of homogeneous information among registries and ethical and data protection regulations that often inhibit the exchange of patient data. Nevertheless, the methodological approach proposed to merge the data appears to be a successful tool for increasing the number of patients and data for future studies.

A possible limitation to our study is that a relevant part of clinical information comes from retrospective data with no efficacy results available. Nevertheless, as pointed out in Additional file 2, retrospective data in Pharmachild were mostly overlapping with prospective data, thus supporting the validity of these safety findings. This limitation becomes crucial when we consider efficacy data, which can be provided only by the prospective analysis. For this reason, further work in the future will be focused on these patients in order to advance the use of JIA drugs through the study of the Pharmachild population. Future analytical work will also have to report accumulated patient years of treatment for each of the registries.

## Conclusions

This article is the first attempt to present a very large sample of data on JIA patients from different national and international registries and represents the first proposal for sharing of data from national and international registries as the most powerful tool for future analysis of safety and effectiveness, with the aim to address important questions on current daily practice in pediatric rheumatology.

## Additional files

Additional file 1: Figure with the data flow. Data flow from individual sites to the PRINTO coordinating center. (PDF 161 kb)

Additional file 2: Table with total number of AEs by MedDRA SOC for retrospective and prospective visits in the Pharmachild registry. Data are absolute numbers and frequencies (percentage) of AEs. SOC are ordered by decreasing frequencies for retrospective AEs. Abbreviations: *AE* adverse event, *MedDRA* Medical Dictionary for Regulatory Activities, *SOC* system organ class. (DOCX 18 kb)

## Abbreviations

AE: Adverse event
BiKeR: Biologics in Pediatric Rheumatology Registry
CRF: Case report form
DMARD: Disease-modifying anti-rheumatic drug
ESI: Events of special interest
ETN: Etanercept
FDA: US Food and Drug Administration
ILAR: International League of Associations for Rheumatology
JADAS: Juvenile Arthritis Disease Activity Score
JIA: Juvenile idiopathic arthritis
JUMBO: Juvenile arthritis MTX/Biologics long-term Observation
MedDRA: Medical Dictionary for Regulatory Activities
MTX: Methotrexate
Pharmachild: Pharmacovigilance in JIA patients treated with biologic agents and/or MTX
PRINTO: Paediatric Rheumatology International Trials Organisation
PRO: Patient-reported outcome
RF: Rheumatoid factor
SOC: System organ class
TNF: Tumor necrosis factor
