# Supplementary figures and images for: Pharmacovigilance in juvenile idiopathic arthritis patients treated with biologic or synthetic drugs: combined data of more than 15,000 patients from Pharmachild and national registries
Source: Arthritis Res Ther. 2018 Dec 27;20:285. doi: 10.1186/s13075-018-1780-z (PMC6307151; doi:10.1186/s13075-018-1780-z)

Additional File 1: Data flow from the individual site to the PRINTO coordinating centre.

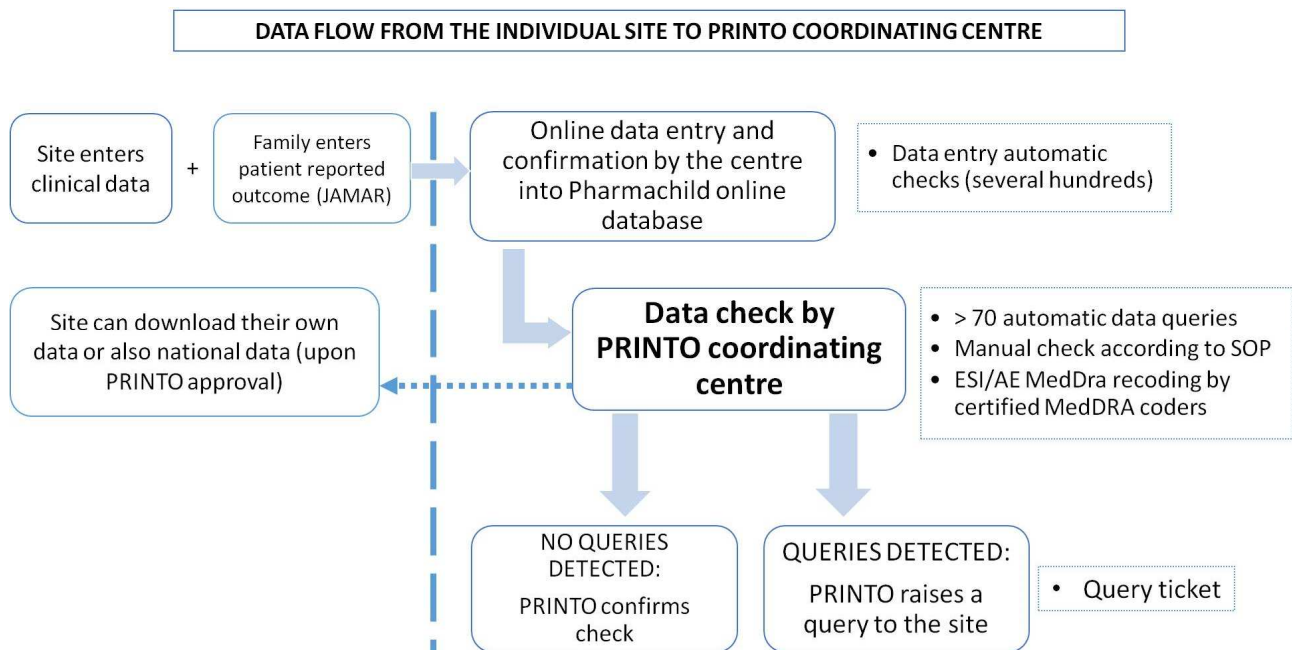

Supplement: Supplementary file 1 — Figure with the data flow. Data flow from individual sites to the PRINTO coordinating center. (PDF 161 kb) [file 13075_2018_1780_MOESM1_ESM.pdf]
