# Supplementary material for: Pharmacovigilance in juvenile idiopathic arthritis patients treated with biologic or synthetic drugs: combined data of more than 15,000 patients from Pharmachild and national registries
Source: Arthritis Res Ther. 2018 Dec 27;20:285. doi: 10.1186/s13075-018-1780-z (PMC6307151; doi:10.1186/s13075-018-1780-z)
Supplement: Supplementary file 2 — Table with total number of AEs by MedDRA SOC for retrospective and prospective visits in the Pharmachild registry. Data are absolute numbers and frequencies (percentage) of AEs. SOC are ordered by decreasing frequencies for retrospective AEs. Abbreviations: AE adverse event, MedDRA Medical Dictionary for Regulatory Activities, SOC system organ class. (DOCX 18 kb) [file 13075_2018_1780_MOESM2_ESM.docx]

Additional File 2: Table with the total number of AEs by MEDdra SOC for retrospective and prospective visits in the Pharmachild registry. Data are absolute numbers and frequencies (%) of AEs. SOC are ordered by decreasing frequencies for retrospectiveAEs.

| Adverse events | **Retrospective**  **N=4123** | **Prospective**  **N=1050** |
| --- | --- | --- |
| Infections and infestations | 1137 (27.6%) | 386 (36.8%) |
| Gastrointestinal disorders | 489 (11.9%) | 106 (10.1%) |
| Injury, poisoning and procedural complications | 277 (6.7%) | 48 (4.6%) |
| Blood and lymphatic system disorders | 253 (6.1%) | 38 (3.6%) |
| Investigations | 245 (5.9%) | 40 (3.8%) |
| Eye disorders | 234 (5.7%) | 36 (3.4%) |
| Hepatobiliary disorders | 199 (4.8%) | 34 (3.2%) |
| Skin and subcutaneous tissue disorders | 189 (4.6%) | 67 (6.4%) |
| General disorders and administration site conditions | 177 (4.3%) | 68 (6.5%) |
| Surgical and medical procedures | 167 (4.1%) | 42 (4.0%) |
| Nervous system disorders | 116 (2.8%) | 35 (3.3%) |
| Musculoskeletal and connective tissue disorders | 114 (2.8%) | 33 (3.1%) |
| Endocrine disorders | 95 (2.3%) | 9 (0.9%) |
| Psychiatric disorders | 85 (2.1%) | 20 (1.9%) |
| Respiratory, thoracic and mediastinal disorders | 76 (1.8%) | 36 (3.4%) |
| Metabolism and nutrition disorders | 62 (1.5%) | 15 (1.4%) |
| Renal and urinary disorders | 62 (1.5%) | 4 (0.4%) |
| Immune system disorders | 30 (0.7%) | 3 (0.3%) |
| Vascular disorders | 26 (0.6%) | 4 (0.4%) |
| Reproductive system and breast disorders | 19 (0.5%) | 7 (0.7%) |
| Cardiac disorders | 17 (0.4%) | 2 (0.2%) |
| Congenital, familial and genetic disorders | 16 (0.4%) | 6 (0.6%) |
| Ear and labyrinth disorders | 12 (0.3%) | 1 (0.1%) |
| Neoplasms benign, malignant and unspecified (incl cysts and polyps) | 11 (0.3%) | 5 (0.5%) |
| Pregnancy, puerperium and perinatal conditions | 9 (0.2%) | 0 |
| Social circumstances | 6 (0.1%) | 5 (0.5%) |
